# Phenotype-Independent Isolation of Interspecies *Saccharomyces* Hybrids by Dual-Dye Fluorescent Staining and Fluorescence-Activated Cell Sorting

**DOI:** 10.3389/fmicb.2019.00871

**Published:** 2019-04-26

**Authors:** Arthur R. Gorter de Vries, Charlotte C. Koster, Susan M. Weening, Marijke A. H. Luttik, Niels G. A. Kuijpers, Jan-Maarten A. Geertman, Jack T. Pronk, Jean-Marc G. Daran

**Affiliations:** ^1^Department of Biotechnology, Delft University of Technology, Delft, Netherlands; ^2^Global Innovation and Research, HEINEKEN Supply Chain B.V., Zoeterwoude, Netherlands

**Keywords:** FACS, *Saccharomyces eubayanus* × *Saccharomyces cerevisiae* hybrids, heterosis, marker-free mating, lager beer brewing, non-GMO

## Abstract

Interspecies hybrids of *Saccharomyces* species are found in a variety of industrial environments and often outperform their parental strains in industrial fermentation processes. Interspecies hybridization is therefore increasingly considered as an approach for improvement and diversification of yeast strains for industrial application. However, current hybridization methods are limited by their reliance on pre-existing or introduced selectable phenotypes. This study presents a high-throughput phenotype-independent method for isolation of interspecies *Saccharomyces* hybrids based on dual dye-staining and subsequent mating of two strains, followed by enrichment of double-stained hybrid cells from a mating population by fluorescence-activated cell sorting (FACS). Pilot experiments on intra-species mating of heterothallic haploid *S. cerevisiae* strains showed that 80% of sorted double-stained cells were hybrids. The protocol was further optimized by mating an *S. cerevisiae* haploid with homothallic *S. eubayanus* spores with complementary selectable phenotypes. In crosses without selectable phenotype, using *S. cerevisiae* and *S. eubayanus* haploids derived from laboratory as well as industrial strains, 10 to 15% of double-stained cells isolated by FACS were hybrids. When applied to rare mating, sorting of double-stained cells consistently resulted in about 600-fold enrichment of hybrid cells. Mating of dual-stained cells and FACS-based selection allows efficient enrichment of interspecies *Saccharomyces* hybrids within a matter of days and without requiring selectable hybrid phenotypes, both for homothallic and heterothallic strains. This strategy should accelerate the isolation of laboratory-made hybrids, facilitate research into hybrid heterosis and offer new opportunities for non-GM industrial strain improvement and diversification.

## Introduction

*Saccharomyces* yeasts are used in various biotechnological industries including beer brewing, wine making, biopharmaceutical protein synthesis, and biofuels production (Balat, 2011; Nielsen, 2013; Marsit and Dequin, 2015; Jansen et al., 2017; Krogerus et al., 2017a). Nine *Saccharomyces* species have currently been described (Hittinger, 2013; Nueno-Palop et al., 2017), which are separated by a post-zygotic barrier that causes interspecies hybrids to be sterile (Naumov et al., 2000; Greig et al., 2002; Pfliegler et al., 2012; Hou et al., 2014). Although *Saccharomyces* hybrids occur in natural contexts such as the guts of wasps (Stefanini et al., 2016), strains with chimeric genomes are most commonly found in domesticated environments (Almeida et al., 2014; Boynton and Greig, 2014). For instance, lager beer is brewed by *S. cerevisiae* × *S. eubayanus* hybrids, collectively indicated as *S. pastorianus* (Libkind et al., 2011), *S. uvarum* × *S. eubayanus* hybrids called *S. bayanus* are used for cider brewing among other applications (Naumov et al., 2001), and various double and triple hybrids between *S. cerevisiae*, *S. kudriavzevii*, and *S. uvarum* play an important role in aroma production during wine fermentation (González et al., 2006). In addition, interspecies hybridization likely contributed to the evolution of domesticated *Saccharomyces* strains by facilitating horizontal gene transfer (Peter et al., 2018). Genetic admixture contributed to the distinct phenotypes of, for instance, cider-fermenting *S. uvarum* strains and wine-fermenting *S. cerevisiae* strains (Naumova et al., 2011; Dunn et al., 2012).

The genomes of hybrids from different *Saccharomyces* species have been shown to act synergistically, a phenomenon called ‘heterosis’ or ‘hybrid vigor,’ in which a hybrid performs better than either of its parents in specific environments (Rainieri et al., 2006; Belloch et al., 2008; Querol and Bond, 2009; Tronchoni et al., 2009). Heterosis is a complex phenomenon, involving copy number effects, interactions between different dominant and recessive alleles, and epistatic interactions (Shapira et al., 2014). Hybrid physiology largely depends on the specific parental strains (Mertens et al., 2015; Krogerus et al., 2017b). While some traits such as cryotolerance or flocculation appear to be completely inherited from one of the parental strains (Coloretti et al., 2006; Hebly et al., 2015), hybrids can also show phenotypes intermediary to their parental strains, as has been demonstrated for production of flavor compounds and other metabolites (Bellon et al., 2011; Krogerus et al., 2015).

*Saccharomyces* hybrids have been generated in the laboratory by crossing strains from different species (Banno and Kaneko, 1989). By analogy to the chimeric hybrids used for industrial applications, laboratory hybridization can yield strains with novel or improved properties for industrial applications. For instance, laboratory-made *S. cerevisiae* × *S. eubayanus* hybrids displayed increased cold tolerance, faster oligosaccharide consumption, different flavor profiles, higher fermentation rates and higher ethanol titres than their parental strains (Steensels et al., 2014a; Hebly et al., 2015; Krogerus et al., 2015). Pioneering studies on reconstruction of naturally-occurring hybrids have inspired the generation of hybrids from novel combinations of species, such as *S. cerevisiae × S. paradoxus* hybrids (Bellon et al., 2011), *S. cerevisiae × S. mikatae* hybrids (Bellon et al., 2013; Nikulin et al., 2017), *S. cerevisiae × S. arboricola* hybrids (Nikulin et al., 2017), and *S. cerevisiae × S. uvarum* hybrids (Masneuf-Pomarède et al., 2002; Bellon et al., 2015; Lopandic et al., 2016). Their phenotypic diversity showed promise for applications ranging from the fermented beverage industry to the production of biofuels (Masneuf-Pomarède et al., 2002; Steensels et al., 2014b; Nikulin et al., 2017; Peris et al., 2017; Nikulin et al., 2018).

Analogous to intra-species mating, interspecies hybridization occurs either by mating haploid cells of opposite mating type, or by rare mating based on spontaneous mating-type switching caused by loss of heterozygosity at the *MAT* locus (Gunge and Nakatomi, 1972). However, interspecies hybridization occurs at a relatively low rate; reported hybridization frequencies range from 1.5 to 3.6% for mass mating of spores (Mertens et al., 2015; Krogerus et al., 2016) to frequencies as low as 1 × 10^−6^ to 1 × 10^−8^ for mass mating of cells dependent on rare mating (Gunge and Nakatomi, 1972; Krogerus et al., 2016). While the efficiency of interspecies mating can be improved by genetic modification (GM) techniques, for example by overexpression of HO-endonuclease (Alexander et al., 2016) or by the use of spore micromanipulation (Naumov, 1996), isolation of *bona fide* hybrids from mating cultures remains necessary.

When parental strains have different selectable phenotypes, hybrids can be isolated by transferring the mating culture to conditions requiring both phenotypes for growth. Selectable phenotypes such as auxotrophies can either occur naturally (Sato et al., 2002; Fernández-González et al., 2015; Magalhães et al., 2017), or they can be obtained by mutagenesis and/or laboratory evolution under conditions favoring auxotrophic strains (Boeke et al., 1987; Scannell et al., 2011; Pérez-Través et al., 2012; Krogerus et al., 2015). However, generation of auxotrophic mutants is time- and labor-intensive (Alexander et al., 2016) and can be further complicated by the polyploid or aneuploid nature of many industrially-relevant *Saccharomyces* strains (Pérez-Través et al., 2012; Gorter de Vries et al., 2017b). Alternatively, selectable phenotypes such as antibiotic resistance can be introduced using GM techniques (Jimenez and Davies, 1980; Goldstein and McCusker, 1999; Piotrowski et al., 2012; da Silva et al., 2015; Hebly et al., 2015). However, industrial strains can be resilient to GM, and customer acceptance and legislation issues still largely preclude use of GM technology for applications in the food and beverages industry (Wunderlich and Gatto, 2015; Gorter de Vries et al., 2017a).

Fluorescence-activated cell sorting (FACS) can be used to isolate fluorescent cells from populations, even if they occur at extremely low frequencies (Cormack et al., 1996). By labeling each parental strain with a fluorescent dye, FACS has previously been used to sort mated *Saccharomyces cerevisiae* cells from their mating culture, resulting in a threefold enrichment of mated cells (Bell et al., 1998). Although a threefold enrichment would not be sufficient to isolate interspecies hybrids from a mating culture, this early study raised the question whether it might be possible to sufficiently modify staining, mating, and FACS procedures to accomplish this goal. To address this question, we explored a method to isolate interspecies *Saccharomyces* hybrids based on dual fluorescent labeling of parental strains and subsequent FACS-based selection of double-stained cells, without any dependency on any selectable phenotypes. After reproducing the isolation of intra-species *S. cerevisiae* crosses, we optimized isolation of interspecies *S. cerevisiae* × *S. eubayanus* hybrids using strains with selectable phenotypes. The resulting method was then tested for phenotype-independent isolation of *S. cerevisiae* × *S. eubayanus* hybrids.

## Materials and Methods

### Strains, Media, and Cultivation

*Saccharomyces cerevisiae* and *S. eubayanus* strains used in this study are listed in Table 1. Strains were routinely grown in complex medium (YP), containing 10 g L^−1^ yeast extract and 20 g L^−1^ peptone, supplemented with 20 g L^−1^ glucose for YPD, and with 20 g L^−1^ trehalose for YPT. Synthetic medium (SM) containing 20 g L^−1^ glucose, 3 g L^−1^ KH_2_PO_4_, 5.0 g L^−1^ (NH_4_)_2_SO_4_, 0.5 g L^−1^ MgSO_4_⋅7H_2_O, 1 mL L^−1^ of a trace element solution and 1 mL L^−1^ of a vitamin solution, was prepared as described previously (Verduyn et al., 1992), and the pH was set to 6.0 using 2 M KOH. Presence of the KanMX marker cassette was selected for in SM+G418: SM supplemented with 0.2 g L^−1^ of G418 (Invitrogen, Carlsbad, CA, United States) in which (NH_4_)_2_SO_4_ was replaced by 1 g L^−1^ monosodium glutamate (Cheng et al., 2000). For solid media, 20 g L^−1^ agar was added to media. Strains were grown in 500 mL round-bottom shake flasks with 100 mL medium at 200 RPM in an Innova 44 incubator shaker (Eppendorf, Nijmegen, Netherlands). Cultures of *S. cerevisiae* and *S. eubayanus* were grown at 30°C and 20°C, respectively. Liquid sporulation medium contained 20 g L^−1^ potassium acetate and its pH set to 7.0 using acetic acid (Bahalul et al., 2010). Frozen stocks were prepared by addition of glycerol (30% v/v) to exponentially growing shake-flask cultures, after which 1-mL aliquots were aseptically stored at −80°C.

**Table 1:** *Saccharomyces* strains used in this study.

Name	Species	Parental strain(s)	Relevant genotype	Origin
CEN.PK113-5A	*S. cerevisiae*	–	*MATa URA3 his3-Δ1 leu2-3,112 trp1-289*	Entian and Kötter, 2007
IMK439	*S. cerevisiae*	CEN.PK113-1A	*MATα HIS3 LEU2 TRP1 ura3*Δ*::KanMX*	González-Ramos et al., 2013
IMK440	*S. cerevisiae*	CEN.PK113-7D	*MATa HIS3 LEU2 TRP1 ura3*Δ*::KanMX*	González-Ramos et al., 2013
CEN.PK122	*S. cerevisiae*	–	*MATa/MATα URA3/URA3*	Entian and Kötter, 2007
CBS 12357	*S. eubayanus*	–	*MATa/MATα*	Libkind et al., 2011
CEN.PK113-7D	*S. cerevisiae*	–	*MATa*	Entian and Kötter, 2007; Salazar et al., 2017
IMS0408	*S. eubayanus × S. cerevisiae*	CBS 12357 × IMK439	*MATa/MATα SeubURA3/Scura3*Δ*::KanMX*	Hebly et al., 2015
CDFM21L.1	*S. eubayanus*	–	*MATa/MATα*	Kindly provided by F.-Y. Bai, Chinese Academy of Sciences (Bing et al., 2014)
Ale28	*S. cerevisiae*	–	*MATa/MATα*	Kindly donated by HEINEKEN Supply Chain, Zoeterwoude, Netherlands
IMX1471	*S. cerevisiae*	IMK439 × IMK440	*MATa/MATα ura3*Δ*::KanMX/ura3*Δ*::KanMX*	This study
IMH001	*S. eubayanus × S. cerevisiae*	CBS 12357 × CEN.PK113-7D	*MATa/MATα*	This study
IMH002	*S. eubayanus × S. cerevisiae*	CBS 12357 × CEN.PK113-7D	*MATa/MATα*	This study
IMH003	*S. eubayanus × S. cerevisiae*	CDFM21L.1 × Ale28	*MATa/MATα*	This study
IMH004	*S. eubayanus × S. cerevisiae*	CDFM21L.1 × Ale28	*MATa/MATα*	This study
IMH005	*S. eubayanus × S. cerevisiae*	CDFM21L.1 × Ale28	*MATa/MATα*	This study
IMH006	*S. eubayanus × S. cerevisiae*	CDFM21L.1 × Ale28	*MATa/MATα*	This study
IMH007	*S. eubayanus × S. cerevisiae*	CDFM21L.1 × Ale28	*MATa/MATα*	This study

### Sporulation, Spore Isolation, and Germination

Sporulation was performed by aerobic incubation at 20°C during at least 72 h on sporulation medium. Presence of asci was verified using phase-contrast microscopy at a magnification of 400x. Spores were isolated as described by Herman and Rine (1997) with minor modifications. In short, spores were pelleted (1000 *g*, 5 min), resuspended in softening buffer (10 mM dithiothreitol, 100 mM Tris-SO_4_, pH set to 9.4 with H_2_SO_4_) and incubated at 30°C for 10 min. Cells were then washed using demineralized water, resuspended in spheroplasting buffer [2.1 M sorbitol, 10 mM KH_2_PO_4_, pH set to 7.2 with 1 M NaOH, 0.8 g L^−1^ zymolyase 20-T (AMS Biotechnology, Ltd., Abingdon, United Kingdom)] and incubated overnight at 30°C. After incubation, the culture was pelleted (1000 *g*, 10 min), washed with demineralized water and resuspended in 0.5% Triton X-100 (Sigma-Aldrich, Zwijndrecht, Netherlands). Spores were then sonicated for 15 s at 50 Hz with an amplitude of 6 μ while kept on ice using a Soniprep 150 (MSE, London, United Kingdom). During initial optimization of the protocol, a short protocol with only the zymolyase-step was also tested. Isolation of spores was confirmed by microscopic inspection as described above. For germination, spores were washed once with YPD and subsequently resuspended in 20 mL YPD to a concentration of approximately 10^6^ cells mL^−1^_._ The germination culture was incubated in a 100 mL round bottom flask at 30°C and 200 RPM for 5 h. A protocol using different incubation times in 2% glucose medium and in YPD was tested during initial optimization of the interspecies mating.

### Staining of *Saccharomyces* Cultures

For staining, CellTrace^TM^ Violet, CellTrace^TM^ CFSE and CellTrace^TM^ Far Red fluorescent dyes (Thermo Fisher Scientific, Waltham, MA, United States) were prepared according to the manufacturers’ recommendations. Cultures were stained with 2 μL CellTrace^TM^ dye per mL culture and incubated overnight in the dark at 12°C and 200 RPM. Stained cultures were washed twice with YP medium, as remaining unbound dye molecules would bind to the amide groups in yeast extract and peptone.

### Intra-Species Mating

Heterothallic haploid parental strains were propagated until mid-exponential phase. The cultures of two parental strains were washed and diluted in sterile Isoton II (Beckman Coulter, Woerden, NL, United States) to a final cell density of approximately 10^6^ cells mL^−1^, stained with CellTrace^TM^ Violet or CellTrace^TM^ CFSE and washed to remove unbound stain. 100 μL of each stained culture was transferred to an Eppendorf tube and centrifuged briefly (2000 *g*, 1 min) to increase proximity of the cells for more efficient mating. Subsequently, the mating culture was statically incubated up to 42 h at 12°C in the dark until FACS analysis. Longer incubation and higher temperatures yielded significant dilution of staining due to cell division.

### Interspecies Mating and Rare Mating

Diploid parental strains and heterothallic haploid parental strains were propagated until mid-exponential phase. Haploid parental cells from homothallic strains were obtained via sporulation, spore isolation and germination. Cells were washed and diluted in sterile Isoton II (Beckman Coulter) to a final cell density of approximately 10^6^ cells mL^−1^, stained with CellTrace^TM^ Violet or CellTrace^TM^ CFSE, and washed to remove unbound stain, as described. For rare mating, a final cell density of approximately 2 × 10^7^ cells mL^−1^ was used and cells were stained with CellTrace^TM^ Far Red or CellTrace^TM^ CFSE. 100 μL of each stained culture was transferred to an Eppendorf tube and centrifuged briefly (2000 *g*, 1 min) to increase proximity of the cells for more efficient mating. Subsequently, the mating culture was statically incubated up to 30 h at 12°C in the dark until FACS analysis. Longer incubation and higher temperatures yielded significant dilution of staining due to cell division.

### FACS Analysis and Sorting

Cultures for FACS analysis and sorting were diluted in sterile Isoton II and vortexed thoroughly to disrupt cell aggregates. For rare mating, 50 mM EDTA was added to disrupt cell aggregates formed by flocculation. The cultures were analyzed on a BD FACSAria^TM^ II SORP Cell Sorter (BD Biosciences, Franklin Lakes, NJ, United States) equipped with 355, 445, 488, 561, and 640 nm lasers and a 70 μm nozzle, and FACSFlow^TM^ sheath fluid (BD Biosciences). Correct cytometer performance was evaluated prior to each experiment by running a Cytometer Setup & Tracking cycle using a CS&T bead kit (BD Biosciences) for calibration. Drop delay for sorting was determined by running an Auto Drop Delay cycle using Accudrop Beads (BD Biosciences). CellTrace^TM^ Violet fluorescence was excited by the 355 nm laser and emission was detected through a 450 nm bandpass filter with a bandwidth of 50 nm. CellTrace^TM^ CFSE was excited by the 488 nm laser and emission was detected through a 545 nm bandpass filter with a bandwidth of 30 nm. CellTrace^TM^ Far Red was excited by the 640 nm laser and emission was detected through a 780 nm bandpass filter with a bandwidth of 60 nm. Morphology of the cells was analyzed by plotting forward scatter (FSC) against side scatter (SSC). Fluorescence of mating cultures was analyzed on either a CFSE versus Violet or a CFSE versus Far Red plot. Prior to sorting, at least 10^5^ events were analyzed. Sorting regions (‘gates’) were set on these plots to determine the types of cells to be sorted. Gated single cells were sorted in 96-well microtiter plates containing YPD using a “single cell” sorting mask (0/32/16), and the plates were incubated at RT for 2 days. When cells with selectable phenotypes were used, the fraction of mated cells was determined by replica-plating to 96-well plates with selective medium (SM or SM+G418), using an ethanol-flame sterilized 96-pin replicator. FACS data were analyzed using FlowJo^®^ software (version 3.05230, FlowJo, LLC, Ashland, OR, United States).

### Determination of the Fraction of Growing Cells

After FACS sorting, the fraction of growing cells was determined by counting the number of wells in which growth was observed. For populations with low viabilities, up to 1000 cells were sorted per well and Poisson statistics were used to estimate the fraction of growing cells (Dube et al., 2008). The fraction of growing cells was calculated from (P), the fraction of wells containing a colony, (W) the total number of wells and (n), the total number of cells sorted into the wells (Eq. 1).

(1) $$\mathrm{Fraction of growing cells}=\frac{-\ln(1-\text{P})*\text{W}}{\text{n}}$$

### Imaging

Cells were imaged using a Zeiss Axio Imager Z1 (Carl Zeiss AG, Oberkochen, Germany). For fluorescent imaging, cells were excited with a xenon lamp. Fluorescence from CellTrace^TM^ CFSE was imaged through a GFP filter set (Carl Zeiss AG) containing a 470 nm bandpass excitation filter with a bandwidth of 20 and 540 nm emission filter with a bandwidth of 25 nm. CellTrace^TM^ Far Red was imaged through a Cy5 filter set (Carl Zeiss AG) containing a 640 nm bandpass excitation filter with a bandwidth of 30 nm and a 690 nm emission filter with a bandwidth of 50 nm. Images were processed using AxioVision SE64 (Rel. 4.9.1. Carl Zeiss AG) and FIJI (Schindelin et al., 2012).

### Ploidy Determination by Flow Cytometry

For ploidy determination, samples were fixed using ethanol as previously described (Hebly et al., 2015). Staining of cells with SYTOX^®^ Green Nucleic Acid Stain (Invitrogen) was performed as previously described (Haase and Reed, 2002) with some minor modifications. Cells were washed in 50 mM Tris-HCl (pH 7.5) and resuspended in 100 μL RNase solution (1 mg/mL RNase A in 50 mM Tris-HCl). 100 μL of cells was added to 1 mL of SYTOX^®^ Green solution. When processing large numbers of samples, a high-throughput protocol in 96-well microtiter plates was used with a PIPETMAN^®^ M multichannel electronic pipette (Gilson, Middleton, WI, United States). In this modified protocol, 100 μL sample was fixated by adding 150 μL 70% ethanol and in the final step 20 μL sample was added to 180 μL SYTOX^®^ Green solution. An unstained control was included along with every sample. Fluorescence of the samples was measured on a BD Accuri^TM^ C6 CSampler Flow Cytometer (BD Biosciences). The fluorophore was excited with the 488 nm laser of the flow cytometer and emission was detected through a 533 nm bandpass filter with a bandwidth of 30 nm. Ploidy data was analyzed using FlowJo^®^ software (version 3.05230, FlowJo).

### Identification of Interspecies Hybrids by PCR

The presence of genetic material from *S. cerevisiae* and from *S. eubayanus* and the mating type of potential hybrids was verified by PCR using DreamTaq PCR Mastermix (Life Technologies), as described previously (Hebly et al., 2015). To ensure that single cells isolates were tested, sorted dual-stained cells were grown in YPD and a second FACS step was used to sort a single cell from each culture. DNA from the single cell isolate cultures was released by boiling 2 μL of a liquid culture in 2 μL of NaOH for 15 min at 99°C. The *S. cerevisiae*-specific *MEX67* gene was amplified using primers 8570 and 8571 (Supplementary Table 1) and the *S. eubayanus*-specific *FSY1* gene was amplified using primers 8572 and 8573 (Muir et al., 2011; Pengelly and Wheals, 2013). The mating type was determined by amplifying *MAT*-loci, using primers 11, 12, and 13 (Huxley et al., 1990). PCR products were separated on a 2% (w/v) agarose gel in 0.5X TBE buffer (45 mM Tris-borate, 1 mM EDTA, pH 8).

## Results

### Isolating Intra-Species Hybrids From a Mating Culture Using FACS

**FIGURE 1:**
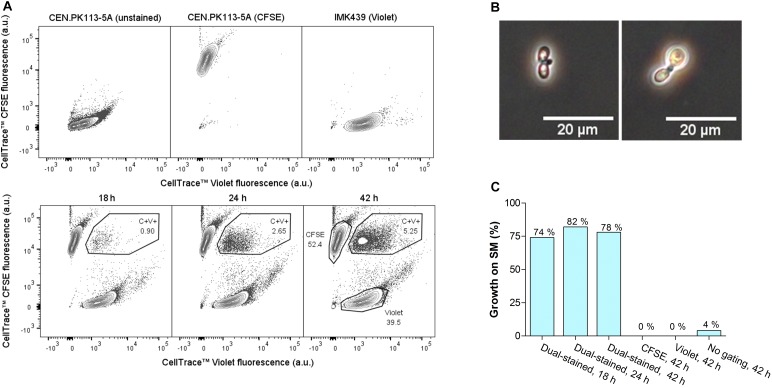
Intra-species mating of *S. cerevisiae* strains CEN.PK113-5A (*MATa URA3 his3*-Δ*1 leu2-3,112 trp1-289*) and IMK439 (*MATα HIS3 TRP1 LEU2 ura3*Δ*::KanMX*). **(A)** Fluorescence contour plots of unstained CEN.PK113-5A, CEN.PK113-5A stained with CellTrace^TM^ CFSE, IMK439 stained with CellTrace^TM^ Violet, and of the mating culture after 18, 24, and 42 h. The indicated gated areas were used for sorting cells, event rates of each gate are indicated as a percentage of total cell counts. **(B)** Microscope image (400×) of zygotes sorted from the double-stained population (C+V+) after 42 h of mating. **(C)** Percentage of cells able to grow on synthetic medium without auxotrophy-complementing supplements in different populations sorted by FACS, as indicated in **(A)**.

A functional protocol for dual staining of parental strains, mating and FACS-based sorting of double-stained cells was developed using the heterothallic haploid *S. cerevisiae* strains CEN.PK113-5A (*MATa*, His^−^, Lys^−^, Trp^−^) and IMK439 (*MAT*α, Ura^−^). Due to their complementary auxotrophies, the fraction of mated cells could easily be quantified before and after FACS-based selection of double-stained cells by measuring the ability to grow on synthetic medium without histidine, lysine, tryptophan, and uracil. CEN.PK113-5A and IMK439 were stained using the commercially-available fluorescent CellTrace^TM^ dyes CFSE and Violet, respectively. These dyes covalently bind to amine groups and thereby irreversibly label the parental cells (Filby et al., 2015). Mated cells should then be identifiable by the presence of fluorescent material from both parental strains. Efficient staining of the parental strains was confirmed for both dyes using flow cytometry (Figure 1A). To minimize dilution of the dye due to cell division, stained cells were mated by co-incubation in YPT medium at 12°C, which resulted in slow growth of *S. cerevisiae*. Flow cytometry of the mating culture indicated a progressive increase of the incidence of double-stained cells, from 0.90% after 18 h to 5.25% after 42 h (Figure 1A). Approximately 10% double-stained cells exhibited a morphology (Figure 1B) characteristic of *Saccharomyces* zygotes (Herskowitz, 1988). To determine the fraction of mated cells, cells from the total population and from the double-stained population were sorted on SM using FACS. Only 4% of the total population was able to grow on SM, while 74–82% of double-stained cells grew on this medium, indicating a 20-fold enrichment of mated cells in the double-stained population (Figure 1C).

### Isolation of Interspecies Hybrids From a Mating Culture Using FACS

**FIGURE 2:**
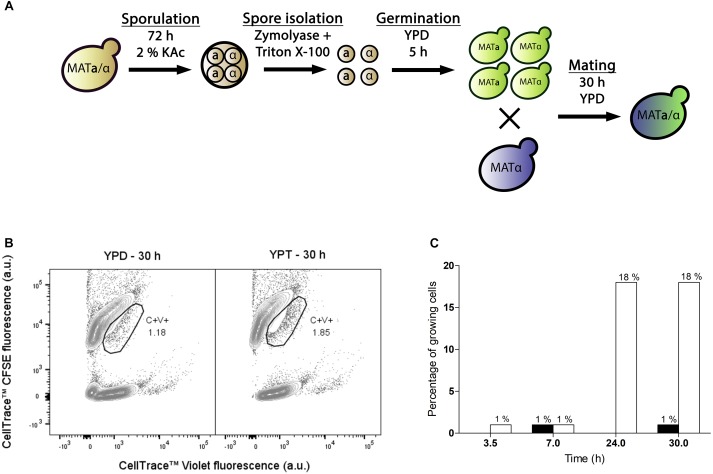
Optimization of interspecies hybridization between haploid *S. eubayanus* and *S. cerevisiae* strains. **(A)** Overview of the optimized protocol for interspecies spore-to-cell mating. **(B)** Fluorescence contour plots of mating cultures of stained CBS 12357 spores and IMK439 cells after 30 h of mating on YPD and YPT. The gated areas were used for sorting cells, event rates of each gate are indicated as a percentage. **(C)** Percentages of cells in the double-stained population able to grow on SM+G418 after 3.5, 7, 24, and 30 h of incubation on YPT (black) and YPD (white). Mating on YPT was only assessed at 7 and 30 h.

The developed protocol was applied to obtain interspecies hybrids between *S. eubayanus* strain CBS 12357 and *S. cerevisiae* strain IMK439 (*MATα, ura3*Δ*::KanMX*). Hybrids of these strains can be easily identified due to combined uracil prototrophy and resistance to the antibiotic G418. As CBS 12357 is a homothallic strain it was sporulated prior to staining and mating. To limit a bias toward self-mating of sister spores, a protocol for digestion of the ascus sack was developed based on a combined treatment with the surfactant Triton X-100 and zymolyase (Herman and Rine, 1997) (Supplementary Figure 1). When staining isolated spores of *S. eubayanus* CBS 12357, approximately half of the population was not fluorescently labeled after staining and incubation (Supplementary Figure 2). As the dye may not be able to penetrate the spore cell wall, the observed loss of staining could be due to loss of bound fluorophores during germination, when the spore cell wall is lost. To allow for efficient germination of spores while minimizing cell division prior to staining, a 5 h incubation on YPD at 30°C was implemented (Supplementary Figure 3). Using a protocol that included these optimizations, germinated spores of *S. eubayanus* CBS 12357 stained with CellTrace^TM^ CFSE were mated with haploid cells of *S. cerevisiae* strain IMK439 stained with CellTrace^TM^ Violet dye (Figure 2A). As the lack of necessity for trehalose consumption would broaden the applicability of our method, we mated the cells in YPD as well as on YPT medium at 12°C. The fraction of hybridized cells was monitored during mating by sorting double-stained cells onto YPD and determining the fraction of sorted cells which could grow on selective medium (Figure 2B). After 7 h, 1% of the double-stained population of both mating cultures on YPT and YPD was hybrid (Figure 2C). In contrast to intra-species *S. cerevisiae* mating, mating on YPT yielded no increase in the fraction of hybrids upon prolonged incubation for interspecies mating. However, in YPD, the fraction of hybrids among the double-stained cells increased to 18% after 24 h and remained stable up to 30 h (Figure 2C). In contrast, after 30 h incubation in YPD without sorting, only 0.3% of the total population was able to grow on selective medium. These results indicated that FACS-based sorting of double-stained cells resulted in a 70-fold enrichment of interspecies hybrids by sorting.

### Enrichment of Interspecies Hybrids Without Selectable Phenotypes

**FIGURE 3:**
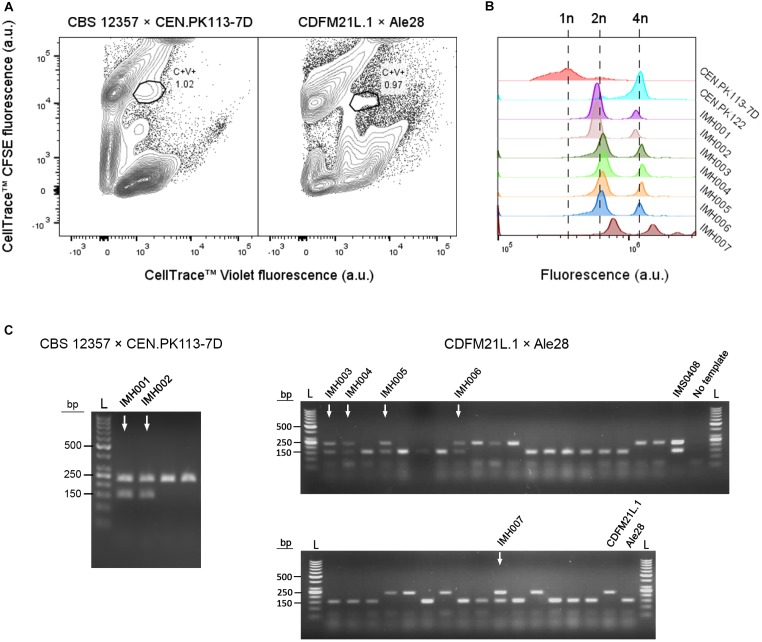
Enrichment of interspecies hybrids without selectable phenotypes from CBS 12357 (*S. eubayanus*, sporulated) and CEN.PK113-7D (*MATa*) and from CDFM21L.1 (*S. eubayanus*, sporulated) and Ale28 (*S. cerevisiae*, sporulated). **(A)** Fluorescence contour plots of mating cultures between CBS 12357 (CFSE) × CEN.PK113-7D (Violet) and CDFM21L.1 (CFSE) × Ale28 (Violet). Gated areas were used for sorting cells, event rates of each gate are indicated as a percentage of the total population size. **(B)** Flow cytometric quantification of the genome content of constructed hybrids using SYTOX Green staining. *S. cerevisiae* strains CEN.PK113-7D and CEN.PK122 were used as a haploid and diploid control, respectively. **(C)** Multiplex PCR amplification of the *S. cerevisiae* specific *MEX67* gene (150 bp) and the *S. eubayanus* specific *FSY1* gene (228 bp) in single-cell isolates of the double-stained populations from CBS 12357 × CEN.PK113-7D and CDFM21L.1 × Ale28 mating cultures. For CBS 12357 × CEN.PK113-7D, 4 of the 22 tested isolates are shown. Genomic DNA of *S. cerevisiae* Ale26, *S. eubayanus* CDFM21L.1 and *S. cerevisiae* × *S. eubayanus* IMS0408 were used as controls. Hybrid isolates are indicated by arrows. L: Generuler 50 bp DNA Ladder.

To test applicability of the dual fluorescent staining FACS protocol for isolation of hybrids without selectable genetic markers, spores of *S. eubayanus* CBS 12357 (Libkind et al., 2011) were crossed with the haploid *S. cerevisiae* strain CEN.PK113-7D(*MATa*) (Entian and Kötter, 2007). In parallel, we crossed spores of the Tibetan *S. eubayanus* isolate CDFM21L.1 (Bing et al., 2014) with spores of the ale-brewing *S. cerevisiae* isolate Ale28, which was provided by HEINEKEN Supply Chain. These diploid strains were sporulated and germinated as described previously (Figure 2A). *S. eubayanus* parents were stained with CellTrace^TM^ CFSE, *S. cerevisiae* parents with CellTrace^TM^ Violet, and cells were co-incubated in YPD during 30 h at 12°C. Individual double-stained cells were sorted into 96 well plates containing 100 μL YPD per well, and incubated at 30°C until cultures were fully grown (Figure 3A). To eliminate false positives due to co-sorting of *S. eubayanus/S. cerevisiae* combinations, a single cell from each well was sorted into a second 96 well plate containing YPD. After incubation at 30°C, the presence of genetic material from both parents was verified by PCR amplification of the *S. cerevisiae* specific *MEX67* gene and of the *S. eubayanus* specific *FSY1* gene (Muir et al., 2011; Pengelly and Wheals, 2013). For the CBS 12357 × CEN.PK113-7D cross, a band corresponding to *MEX67* and to *FSY1* was observed for 2 of 22 tested single-cell isolates. These isolates were stored as IMH001 and IMH002 (Figure 3C). For the CDFM21L.1 × Ale28 cross, a band corresponding to *MEX67* and to *FSY1* was produced for 5 of 34 tested single-cell isolates, which were stored as IMH003-IMH007 (Figure 3C). To verify if strains IMH001-IMH007 were hybrids and not mixtures of haploid *S. cerevisiae* and *S. eubayanus* cells, the ploidy of the sorted cells was determined by DNA staining using SYTOX Green and flow cytometric analysis. The genome content of IMH001-IMH006 was diploid, whereas IMH007 was aneuploid (Figure 3B), indicating successful mating. Therefore, 9% of tested cells from the mating between CBS 12357 and CEN.PK113-7D and 15% of cells from the mating between CDFM21L.1 and Ale28 were hybrids. These results indicate that fluorescent staining and FACS enable a substantial enrichment of hybrid cells both for laboratory and industrial-relevant strains. A simple PCR protocol was sufficient to identify hybrids after enrichment.

### Enrichment of Interspecies Hybrids by Rare Mating

Polyploidy and aneuploidy are commonly observed in industrial *Saccharomyces* hybrids (González et al., 2006; Querol and Bond, 2009; Peris et al., 2012), and chromosome copy number can play a key role in industrial performance (Krogerus et al., 2016; Gorter de Vries et al., 2017b). The poor sporulation efficiency of many industrial strains can preclude hybridization by conventional mating (Anderson and Martin, 1975). We explored the use of dual fluorescent staining and FACS to enrich hybrids obtained by rare mating by testing combinations of haploid and diploid *S. eubayanus* and *S. cerevisiae* strains. To evaluate low fractions of hybrid cells, we used *S. eubayanus* strains with uracil prototrophy and *S. cerevisiae* strains with uracil auxotrophy and resistance to the antibiotic G418. To obtain a diploid *S. cerevisiae* strain with uracil auxotrophy and resistance to the antibiotic G418, we first crossed IMK439 (*MATα ura*3Δ::KanMX) and IMK440 (*MATa ura3*Δ*::KanMX*) using fluorescent staining and FACS, resulting in IMX1471 (*MATa/MATα, ura3*Δ*::KanMX/ura3*Δ*::KanMX*). The mating types, ploidy, ability to sporulate, uracil prototrophy and G418 resistance of IMX1471 were verified (Supplementary Figure 4). Due to the anticipated low frequency of rare mating, *S. eubayanus* cells were stained with CellTrace^TM^ CFSE and *S. cerevisiae* cells with CellTrace^TM^ Far Red, as these dyes have little spectral overlap (Supplementary Figure 4). In total, three different crosses were made: CBS 12357 (sporulated, 1n) × IMX1471 (2n), CBS 12357 (2n) × IMK439 (1n) and CBS 12357 (2n) × IMX1471 (2n). The frequency of hybrid cells in each mating culture was assessed by plating 2 × 10^8^ cells on SM+G418 plates and counting colonies. In parallel, the mating culture was analyzed by FACS and double-stained cells were sorted and replica-plated to SM+G418 to determine the frequency of hybrid cells after sorting. Due to the low frequency of rare mating, wells were inoculated with 1, 10, or 100 double-stained cells and the fraction of growing cells was calculated using Poisson statistics.

For the CBS 12357 (2n) × IMK439 (1n) cross, the fraction of hybrids in the total population varied between 1.6 × 10^−6^ and 7.2 × 10^−6^ between 24 and 168 h. After sorting, the fraction increased on average by a factor of 590 to between 4.3 × 10^−4^ and 1.3 × 10^−3^ (Table 2). For the CBS 12357 (1n) × IMX1471 (2n) cross, the fraction of hybrids in the total population varied between 3 × 10^−7^ and 1.5 × 10^−6^ between 24 and 168 h. Sorting only yielded a single hybrid after 96 h, at a fraction on 4.3 × 10^−4^, corresponding to a 540-fold enrichment. For the CBS 12357 (2n) × IMX1471 (2n) cross, a single hybrid was observed after 96 h of incubation, corresponding to a rate of 1 × 10^−7^, while no hybrids were identified after sorting. Overall, while rare mating was possible between the haploid and diploid strains, mated cells were present in very low frequencies both in the mating cultures and in the double-stained cells. In theory, fluorescent staining and FACS could be combined with high throughput PCR screening for hybrids in the sorted population. However, hundreds of cells would need to be screened for the diploid CBS 12357 × haploid IMK439 cross, and even more for the other crosses.

**Table 2:** Fraction of hybrid cells after interspecies rare mating between *S. eubayanus* strain CBS 12357 and *S. cerevisiae* strains IMK439 (1n) and IMX1471 (2n) as determined by the ability to grow on SM+G418.

	CBS 12357 × IMK439		CBS 12357 (spores) × IMX1471		CBS 12357 × IMX1471	
	(2n × 1n)		(1n × 2n)		(2n × 2n)	
Mating time (h)	Total population	After sorting	Total population	After sorting	Total population	After sorting
24	–	4.3 × 10^−4^	–	–	–	–
48	4.6 × 10^−6^	2.9 × 10^−3^	8 × 10^−7^	–	–	–
72	–	4.1 × 10^−3^	–	–	–	–
96	7.2 × 10^−6^	4.3 × 10^−3^	8 × 10^−7^	4.3 × 10^−4^	1 × 10^−7^	–
120	1.9 × 10^−6^	1.3 × 10^−3^	9 × 10^−7^	–	–	–
144	1.6 × 10^−6^	4.3 × 10^−4^	3 × 10^−7^	–	–	–
168	4.7 × 10^−6^	3.6 × 10^−3^	1.5 × 10^−6^	–	–	–

*Due to propagation of non-mated cells, the fraction of hybrid cells does not necessarily increase over time. The fraction of hybrids in the total population was determined by plating approximately 2 × 10^8^ cells of the mating culture on SM+G418. For the fraction of hybrid cells in the double-stained population, 1 cell was sorted into 48 wells, 10 cells into 24 wells and 100 cells into 24 wells of a 96-well plate with YPD, which was replica-plated to SM+G418. Fractions of growing cells were calculated using with Poisson statistics. The sign “-” indicates that no hybrids were identified.*

## Discussion

This study presents a new method for the enrichment of interspecies *Saccharomyces* hybrids that does not require parental strains and/or the resulting hybrids to have selectable phenotypes. By dual staining of parental cells with commercially-available fluorescent dyes prior to mating, mated cells could be enriched by up to 600-fold through sorting double-stained cells using FACS. In order to be able to mate homothallic strains, we developed a protocol for sporulation and germination prior to staining. Double-stained subpopulations selected by FACS after application of this protocol contained about 80% mated cells for intra-species crosses and 10–15% of mated cells for interspecies hybridization. By screening sorted double-stained cells using PCR, hybrids were successfully isolated from crosses of both laboratory strains and industrially relevant strains that did not have selectable phenotypes. By circumventing the need of conventional hybridization techniques for pre-existing or engineered selectable phenotypes (Pérez-Través et al., 2012; da Silva et al., 2015; Fernández-González et al., 2015; Krogerus et al., 2015), this method enables the isolation of hybrids from a wide range of strains within just a few days. Interspecies hybrids have been obtained previously by fluorescent staining and protoplast fusion (Katsuragi et al., 1994). However, while protoplast fusion is considered as a GM technique in some countries, hybridization by mating is not, making it suitable for application in the globalized food and beverage industry (Gibson et al., 2017). The use of staining is not problematic for industrial application as it is rapidly lost by dilution during subsequent cell division of the hybrid cells (Filby et al., 2015).

The isolation of interspecies hybrids using fluorescent labeling and FACS provides new opportunities for the use of laboratory-made hybrids for applications such as the production of fermented beverages and biofuels (Bellon et al., 2011, 2015; Mertens et al., 2015; Magalhães et al., 2017; Peris et al., 2017). Since hybrid physiology depends strongly on the combination of parental strains (Mertens et al., 2015; Krogerus et al., 2017b), the possibility to mate strains without any selectable phenotype could widen the phenotypic diversity of laboratory-made hybrids. Germination, staining and/or mating conditions could be optimized to account for traits of specific parental strains such as temperature optima, carbohydrate utilization, and growth kinetics. In the future, mass-mating of more than two fluorescently-labeled parental strains could broaden the phenotypic diversity of obtained laboratory-hybrids. Due to the inability of many industrial strains to produce viable spores (Steensels et al., 2014b), and due to the potential value of higher-ploidy hybrids (Krogerus et al., 2016; Gorter de Vries et al., 2017b), the isolation of interspecies hybrids by rare mating would also be valuable for industrial strain development. While fluorescent labeling and FACS did enable a 600-fold enrichment of interspecies hybrids obtained by rare mating, the isolation of hybrids would require extensive screening due to the high incidence of false positives. The presence of both stains in virtually all sorted cells, as verified by microscopy, indicated that false positives are not due to unwanted co-sorting of single stained cells. Instead, staining may be transferred between cells without resulting in hybrid cells. Due to the covalent binding of stains, such transfer would likely involve transfer of significant amounts of cell components, such as cell membrane or cytoplasm. Such transfer could result from abortive mating or cytoduction, which result in cytoplasmic fusion without nuclear fusion and would likely increase in frequency as mating efficiency decreases (Sigurdson et al., 1981). However, PCR based screening of hundreds of candidates is not impossible in industrial strain improvement programs. Moreover, the development of high throughput methods such as microfluidic lab-on-a-chip setups could further simplify screening after FACS sorting of rare hybrids (Schmitz et al., 2009; Gach et al., 2017).

The genome composition and stability of laboratory hybrids has been extensively researched (Sipiczki, 2018). However, the ability to generate diverse interspecies hybrids using fluorescent labeling could simplify ongoing research on, for example, hybrid-specific phenomena such as heterosis (Shapira et al., 2014; Bernardes et al., 2017), the inheritance of mitochondrial DNA in hybrids (Hsu and Chou, 2017), genome stability and loss of heterozygosity (Smukowski Heil et al., 2017) or hybrid sterility (Greig et al., 2002; Lee et al., 2008). Overall, the interspecies mating procedure presented here may be strongly accelerate industrial strain improvement programs and fundamental research into hybrid yeasts. Moreover, the described method can be complementary with subsequent strain improvement: laboratory evolution can result in admixture of parental genomes, resulting in further adaptation and potentially in improved performance, analogous to the admixed genomes of industrial chimeric strains (Pérez Través et al., 2014; Peris et al., 2017; Gorter de Vries et al., 2019).

## Data Availability

The raw data supporting the conclusions of this manuscript will be made available by the authors, without undue reservation, to any qualified researcher.

## Author Contributions

AG and J-MD conceived the study. AG, CK, SW, and ML designed experimental approaches. CK, SW, and AG performed experimental work. NK and J-MG provided strain ALE28 and supported the project. AG and CK wrote the manuscript with critical input from JP and J-MD. JP and J-MD supervised the study. All authors read and approved the final manuscript.

## Conflict of Interest Statement

AG, CK, NK, J-MG, and J-MD have filed a patent application concerning this work. NK and J-MG are employed by Heineken Supply Chain B.V. (Zoeterwoude, Netherlands). The remaining authors declare that the research was conducted in the absence of any commercial or financial relationships that could be construed as a potential conflict of interest.
