# Supplementary material for: Phenotype-Independent Isolation of Interspecies Saccharomyces Hybrids by Dual-Dye Fluorescent Staining and Fluorescence-Activated Cell Sorting
Source: Front Microbiol. 2019 Apr 26;10:871. doi: 10.3389/fmicb.2019.00871 (PMC6498416; doi:10.3389/fmicb.2019.00871)
Supplement: Supplementary file 1 [file Data_Sheet_1.PDF]

Supplementary Material

Supplementary table 1. Primers used in this study

Primer #	Name	Sequence 5' to 3'	Product size (bp)	Description
8570	Scer F2	GCGCTTTACATTCAGATCCCGAG	150	*S. cerevisiae* specific primers
8571	Scer R2	TAAGTTGGTTGTCAGCAAGATTG		
8572	Seub F3	GTCCCTGTACCAATTTAATATTGCGC	228	*S. eubayanus* specific primers
8573	Seub R2	TTTCACATCTCTTAGTCTTTTCCAGACG		
11	Matuniv	AGTCACATCAAGATCGTTTATGG	MATα: 404<br>MATa: 504	*S. cerevisiae* MAT cassette (α and a)
12	Mat-alpha	GCACGGAATATGGGACTACTTCG		
13	MATa	ACTCCACTTCAAGTAAGAGTTTG		

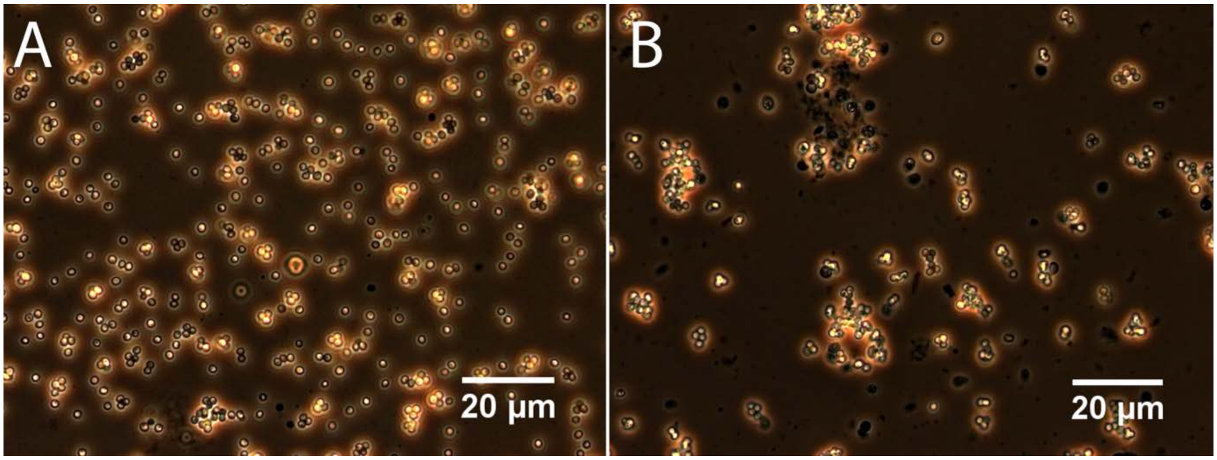

Supplementary figure 1. Microscopy images (400x) of *S. eubayanus* CBS 12357 spores after digestion of the ascus wall. (A) Isolation by zymolyase digestion and treatment with the surfactant Triton X-100 treatment as described by Herman and Rine [54]. (B) Isolation by zymolyase digestion only.

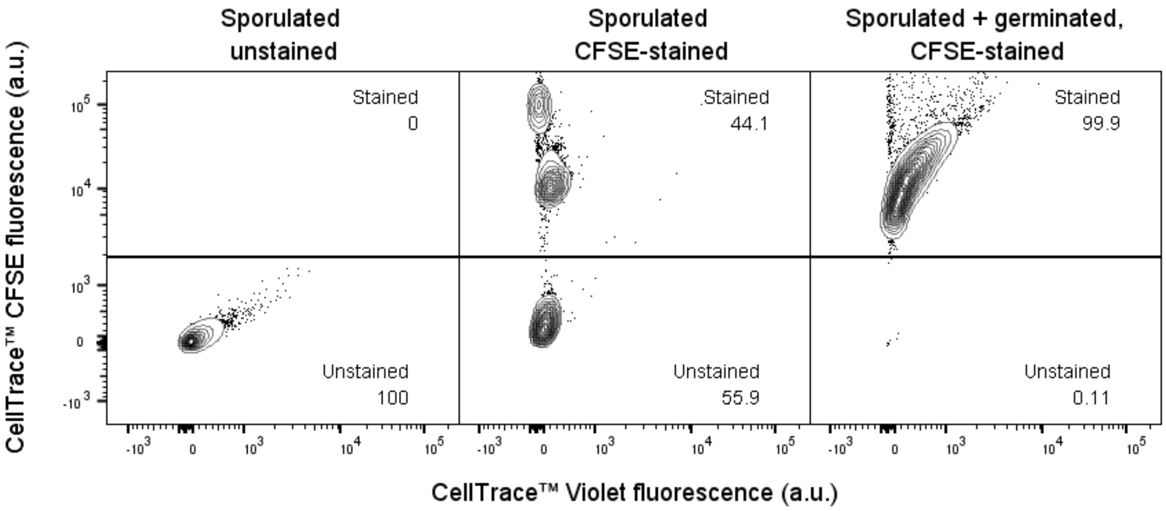

Supplementary figure 2. Fluorescence contour plots of CBS 12357 (*S. eubayanus, wt)* without a stain, stained with CellTrace™ CFSE after sporulation only and stained with CellTrace™ CFSE after sporulation and germination. Event rates of each gate are indicated as a percentage.

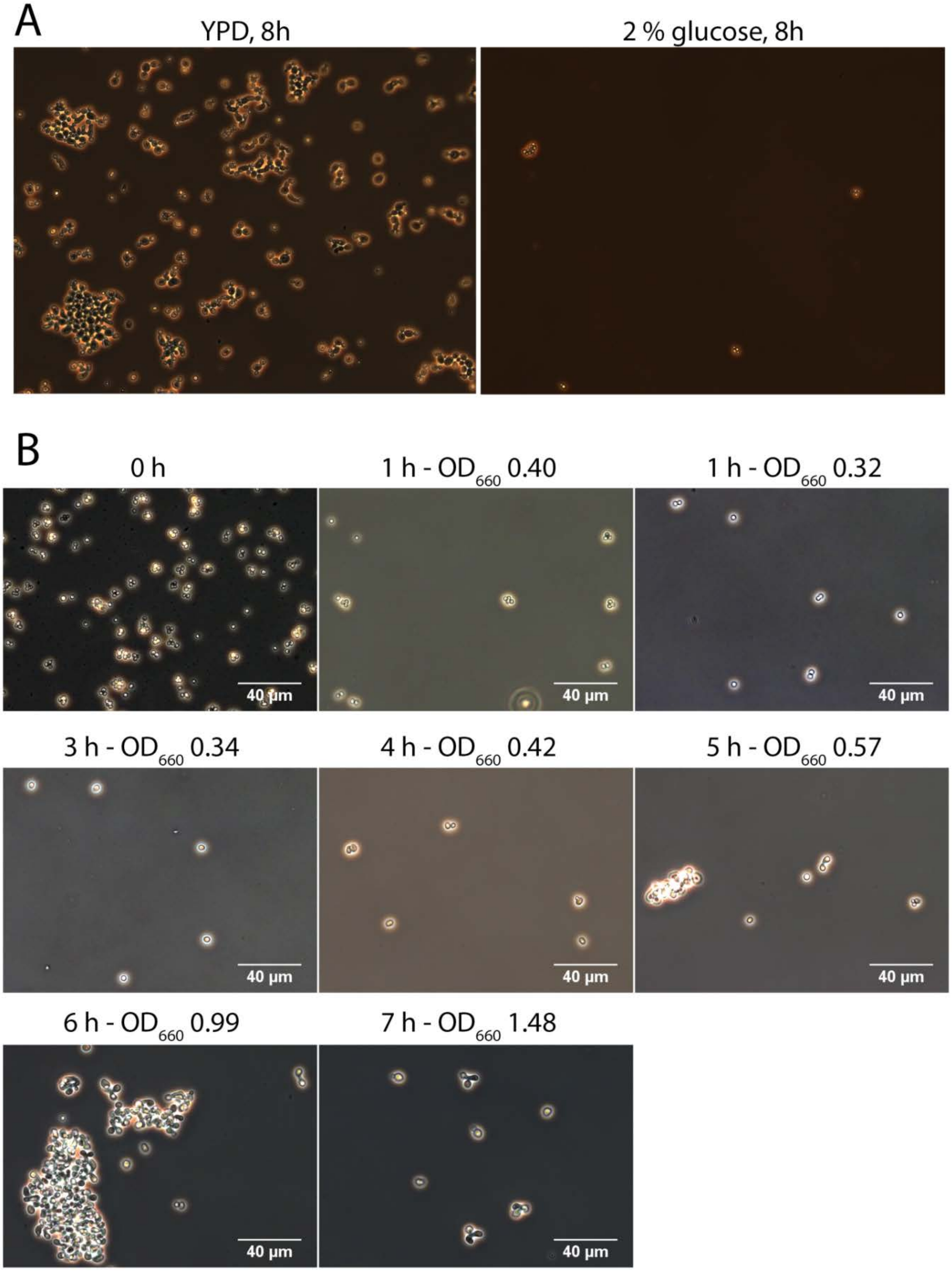

Supplementary figure 3. Microscopy images (400x) during germination of *S. eubayanus* CBS 12357 spores after (A) 8 h of germination on YPD and 2 % glucose and (B) 0 – 7 h of germination on YPD. The OD_{660} of the germination cultures at the time of imaging is indicated.

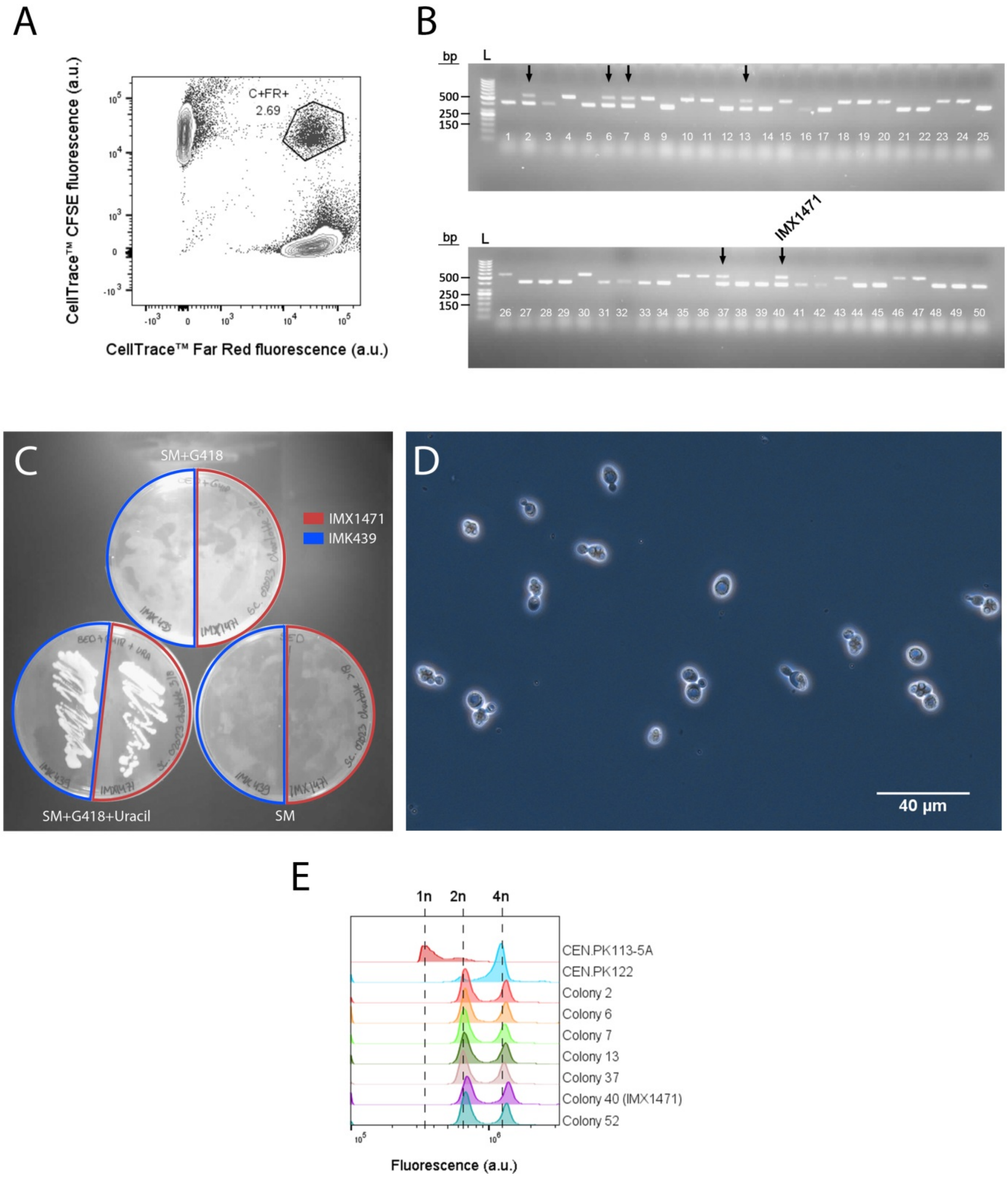

Supplementary figure 4. Construction and validation of IMX1471 by mating IMK439 (*S. cerevisiae*, *MATα ura3Δ::KanMX*) with IMK440 (*S. cerevisiae*, *MATa ura3Δ::KanMX*). (A) Fluorescence contour plot of stained IMK439 (CellTrace™ CFSE) and IMK440 (CellTrace™ Far Red) cells after 30 h of mating on YPD. The gated areas were used for sorting cells, event rates of each gate are indicated as a percentage. (B) Multiplex PCR amplification of the *MATa* and *MATα* mating type cassette in single-cell isolates of the double-stained population from a IMK439 ×

IMK440 mating culture using primers 11, 12 and 13 (Supplementary table 1). Mated cells are indicated by arrows. L: Generuler 50 bp DNA Ladder. (C) Characterization of Uracil prototrophy and G418 resistance of IMK439 and IMX1471 by plating on SM, SM+G418 and SM+G418+Uracil. (D) Microscope image (400x) of IMX1471 after 72 h on sporulation medium. (E) Flow cytometric quantification of the genome content of double-stained cells from the IMK439 × IMK440 mating culture using SYTOX Green staining. CEN.PK113-7D and CEN.PK122 were used as a haploid and diploid control, respectively.
